# Decoding extremophiles: insights from bioinformatics, machine learning, and data-driven approaches

**DOI:** 10.1093/bib/bbag236

**Published:** 2026-05-19

**Authors:** Maria N Chasapi, Nicholas Kontis, Robert Lehmann, Ruqaiya Tasneem, Niketan S Patel, Sumeer A Khan, Xabier Martínez de Morentin, Iro N Chasapi, Eleni Aplakidou, Alexandros Galaras, Lila Aldakheel, Minjing Su, Fotis A Baltoumas, Kasthuri Venkateswaran, Vincenzo Lagani, David Gómez-Cabrero, Jesper Tegnér, Georgios A Pavlopoulos, Alexandre Soares Rosado

**Affiliations:** Institute for Fundamental Biomedical Research, BSRC “Alexander Fleming”, 34 Fleming Str., Vari, 16672, Greece; Biological and Environmental Science and Engineering Division, 4700 King Abdullah University of Science and Technology (KAUST), Thuwal 23955-6900, Saudi Arabia; Biological and Environmental Science and Engineering Division, 4700 King Abdullah University of Science and Technology (KAUST), Thuwal 23955-6900, Saudi Arabia; Biomedical Sciences Division, 4700 King Abdullah University of Science and Technology (KAUST), Thuwal 23955-6900, Saudi Arabia; National Institute of Animal Biotechnology(NIAB), Opp. Journalist Colony, Near Gowlidoddy, Extended Q City Road, Gachibowli, Hyderabad 500032, Telangana, India; Biological and Environmental Science and Engineering Division, 4700 King Abdullah University of Science and Technology (KAUST), Thuwal 23955-6900, Saudi Arabia; Biomedical Sciences Division, 4700 King Abdullah University of Science and Technology (KAUST), Thuwal 23955-6900, Saudi Arabia; Biomedical Sciences Division, 4700 King Abdullah University of Science and Technology (KAUST), Thuwal 23955-6900, Saudi Arabia; Institute for Fundamental Biomedical Research, BSRC “Alexander Fleming”, 34 Fleming Str., Vari, 16672, Greece; Department of Informatics and Telecommunications, National and Kapodistrian University of Athens, Panepistimioupolis, Ilisia, 16122 Athens, Greece; Institute for Fundamental Biomedical Research, BSRC “Alexander Fleming”, 34 Fleming Str., Vari, 16672, Greece; Division of Basic Sciences, University of Crete Medical School, Voutes, Heraklion 70013, Greece; Institute for Fundamental Biomedical Research, BSRC “Alexander Fleming”, 34 Fleming Str., Vari, 16672, Greece; Biological and Environmental Science and Engineering Division, 4700 King Abdullah University of Science and Technology (KAUST), Thuwal 23955-6900, Saudi Arabia; Biological and Environmental Science and Engineering Division, 4700 King Abdullah University of Science and Technology (KAUST), Thuwal 23955-6900, Saudi Arabia; Institute for Fundamental Biomedical Research, BSRC “Alexander Fleming”, 34 Fleming Str., Vari, 16672, Greece; Department of Space Studies, University of North Dakota, Grand Forks 4149 University Avenue, Grand Forks, ND 58202ND 58202-9008, P 701.777.2480, United States; Biomedical Sciences Division, 4700 King Abdullah University of Science and Technology (KAUST), Thuwal 23955-6900, Saudi Arabia; Institute of Chemical Biology, Ilia State University, 3/5 K. Cholokashvili Ave, Tbilisi 0162, Georgia; Biomedical Sciences Division, 4700 King Abdullah University of Science and Technology (KAUST), Thuwal 23955-6900, Saudi Arabia; Biomedical Sciences Division, 4700 King Abdullah University of Science and Technology (KAUST), Thuwal 23955-6900, Saudi Arabia; Unit of Computational Medicine, Department of Medicine, Center for Molecular Medicine, Karolinska University Hospital, Visiongatan 18, SE-171 76 Stockholm, Sweden; Computer, Electrical and Mathematical Sciences and Engineering Division, 4700 King Abdullah University of Science and Technology (KAUST), 23955-6900 Thuwal, Saudi Arabia; Science for Life Laboratory, Karolinska Institutet, Tomtebodavagen 23A, SE-17165 Solna, Sweden; Institute for Fundamental Biomedical Research, BSRC “Alexander Fleming”, 34 Fleming Str., Vari, 16672, Greece; Department of Computational Biology, Mohamed bin Zayed University of Artificial Intelligence (MBZUAI), Building 1B, Masdar City, SE45 05, Abu Dhabi, United Arab Emirates; Biological and Environmental Science and Engineering Division, 4700 King Abdullah University of Science and Technology (KAUST), Thuwal 23955-6900, Saudi Arabia; Biomedical Sciences Division, 4700 King Abdullah University of Science and Technology (KAUST), Thuwal 23955-6900, Saudi Arabia

**Keywords:** extremophiles, cultivation, bioinformatics, metagenomics, multi-omics, machine learning

## Abstract

Life thrives in Earth’s most inhospitable environments, from boiling hydrothermal vents to hypersaline lakes and frozen polar deserts, thanks to the remarkable adaptations of extremophilic microorganisms. The study of these organisms has rapidly evolved from early cultivation-based discoveries to a data-rich discipline powered by advanced omics technologies. This review comprehensively outlines the current landscape and future directions in extremophile research, emphasizing the pivotal role of bioinformatics, machine learning (ML), and data-driven approaches. We begin by charting the evolution of methodologies, from innovative *in situ* cultivation techniques and robust biomolecule extraction protocols to modern multi-omics workflows (metagenomics, transcriptomics, proteomics, and metabolomics) that decode the genetic and functional basis of extremophiles. We then catalogue essential bioinformatics resources and specialized databases critical for annotating extremophile genomes and uncovering their unique adaptive strategies, including protein stabilization and syntrophic metabolic relationships. Finally, we explore the transformative potential of artificial intelligence (AI) and ML in overcoming fundamental challenges in the field. These include predicting the functions of uncharacterized “hypothetical” proteins, identifying novel extremozymes, modeling complex genotype–phenotype relationships, and guiding the targeted engineering of industrially relevant strains. By synthesizing insights across these domains, this review highlights how integrating computational biology and AI is poised to unlock the full biotechnological potential of extremophiles and redefine the boundaries of life itself.

## Introduction

Over the past century, exploration of Earth’s most inhospitable niches has transformed our understanding of where life can persist and how it does so. In 1969, Brock & Freeze isolated the first nonspore-forming thermophile, *Thermus aquaticus*, from a hot spring in Yellowstone Park, USA, demonstrating robust growth near 70°C in both natural and laboratory settings [1]. However, contemporaneous anthropocentrism at the time entrenched a human-centered physiological baseline, characterized by narrow temperature (∼20°C–40°C), pH (6–8), salinity (∼0.1–0.5 M NaCl), and pressure ranges, such that any deviation is labeled as “extreme.” This human notion guided early expeditions away from habitats that appeared only subtly outside these human norms, delaying the discovery of meso- to polyextremophilic microorganisms [2, 3]. Within a few years, Malceroy [3] formalized the concept of organisms that prefer such conditions, naming them “lovers” (“philos” in Greek) of extreme environments. Malceroy introduced the term “extremophiles” to distinguish these (“extreme-loving” organisms) from extremotolerant taxa that merely endure stresses without requiring them.

Meanwhile, a vast deep biosphere, largely invisible at the time, was coming into view. Prokaryotic cells extend down to 5 km beneath continents and several kilometers into the oceanic crust, with recent estimates indicating that 5 × 10^29–1.2 × 10^30 cells inhabit these dark sediments. Considering that this sheer number might even surpass microbes on the surface, extremophiles may well constitute one of the most abundant lifeforms on our planet. Based on recent studies, molecular clocks further suggest that hyperthermophilic and acidophilic lineages diverged early, consistent with an Archean Earth (∼4.0–2.5 Ga) where extremophily may have been the modal lifestyle [4–6].

Today, extremotolerant and extremophilic microbes have been detected under a wide range of temperatures, pH, salinity, desiccation, radiation, pressure, energy, and nutrient-limited conditions, continually redefining the boundaries of conditions under which life can thrive. Extremophiles can persist in these challenging environmental conditions due to a long history of intense adaptations driven by episodic shifts in Earth’s geochemistry and climate [7]. Given that “modern” surface conditions are relatively recent on geological timescales, it is plausible that extremophile strategies helped shape core metabolic processes long before mesophilic niches expanded [8, 9].

**Figure 1:**
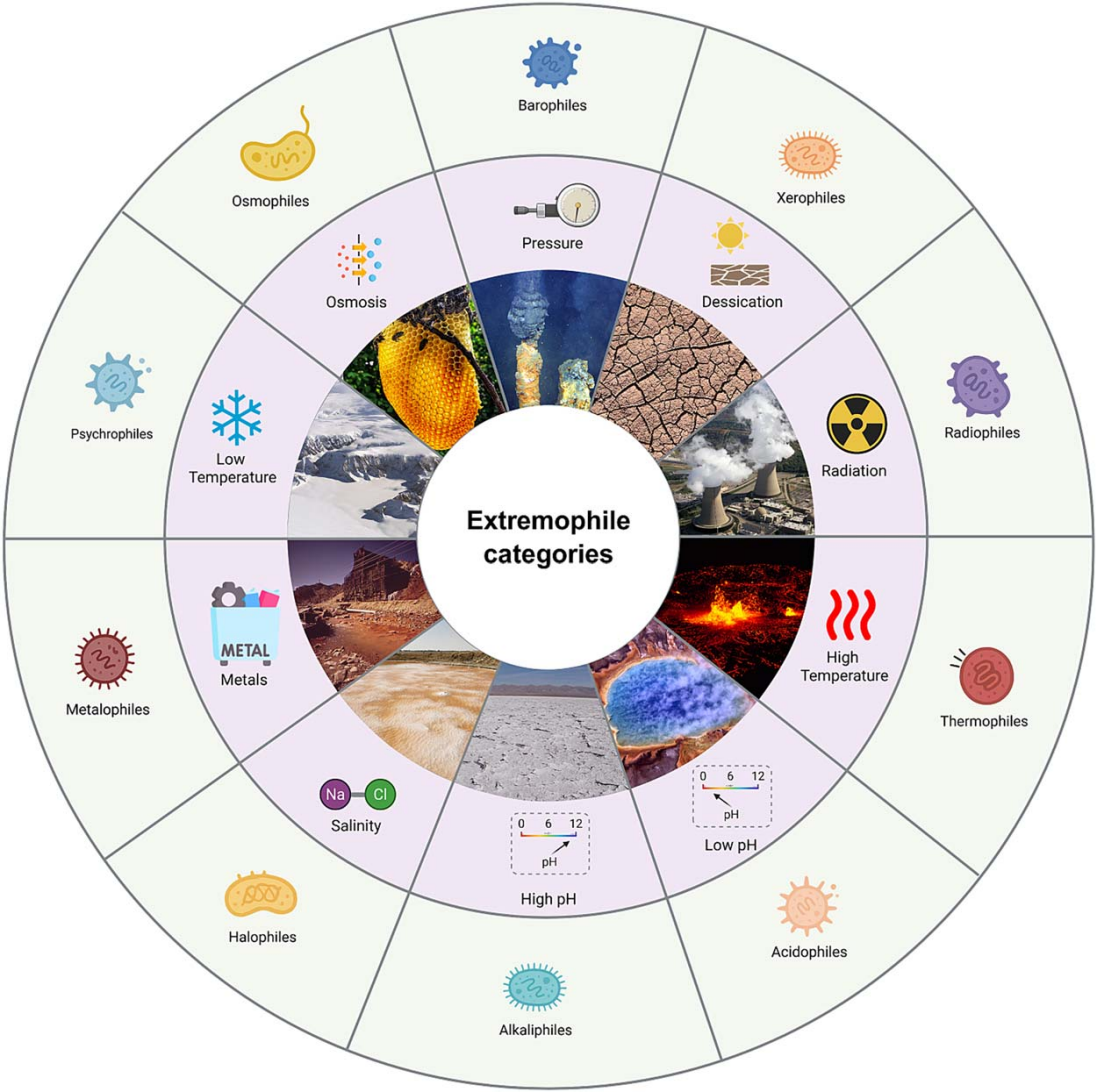
Main categories of extremophiles. This illustration depicts the 10 most common extremophile types, highlighting their defining physicochemical stressors and representative natural environments.

Extremophiles are defined by the physicochemical extremes at which they grow optimally, and many types satisfy more than one criterion (polyextremophiles) (Fig. 1). Thermophiles tend to thrive between temperatures of 50°C and 80°C, while hyperthermophiles exhibit optimum growth at 80°C or higher. They stabilize proteins and membranes via heat-shock chaperones, ionic networks, and archaeal ether lipids. Psychrophiles grow optimally below 15°C with a maximum temperature of 20°C and have even been found to thrive under 0°C. They tolerate low temperatures by using highly unsaturated membranes, cold-active enzymes, antifreeze proteins, and extracellular polysaccharides. Acidophiles prefer a pH below 3 and maintain extreme proton gradients through impermeable membranes and cytoplasmic buffering. In contrast, alkaliphiles tend to grow and thrive at pH values above 9 by exploiting sodium bioenergetics, pH homeostasis transporters, and alkalistable cell walls. Xerophiles thrive at low water activity (<~0.8) by accumulating compatible solutes (trehalose, ectoine) and vitrifying sugars. At the same time, osmophiles tolerate very high osmotic pressures, typically those of concentrated sugars, via the uptake of osmoprotectants and specialized aquaporins. Halophiles require elevated salinity, ranging from moderate (3%–15%) to extreme (15%–30%). They can counterbalance ionic stressors by accumulating potassium and maintaining acidic, salt-stabilized proteomes. Radiophiles withstand intense ionizing and Ultraviolet (UV) radiation (several kilograys) through manganese-based antioxidant systems, robust DNA repair systems, and protective carotenoids. Metallophiles tolerate or utilize high concentrations of metals and metalloids using efflux pumps, chelation, redox transformations, and biomineralization. Barophiles or piezophiles grow best at high hydrostatic pressures (≥10 MPa), with hyperpiezophiles requiring hadal-trench pressures (≥70–100 MPa). They employ pressure-adapted enzymes, piezolytes, and fluidized membranes. Other frequently encountered categories include anaerobes and microaerophiles (oxygen extremes), endoliths inhabiting rock matrices, oligotrophs adapted to chronic nutrient limitation, and polyextremophiles such as cold, hypersaline brine specialists [7, 10, 11] (Fig. 1).

As exploration has accelerated, so too has the catalogue of extreme habitats harboring microbial life. Microorganisms have been identified in sea ice permafrost and polar regions, cold seeps and mud volcanoes, shallow-water hydrothermal vents, fumaroles, hyperacidic lakes and volcanoes, deserts and arid environments, acid mine drainage, deep-sea anoxic lakes and brines, serpentinizing environments, deep-sea sediments and trenches, soda lakes and hypersaline lakes, nuclear contaminated sites, and ophiolites. Given this variety of environments, microbial life can be found virtually anywhere where liquid water is available. The continuous discovery of microbial life in previously unexplored extremes demonstrates the highly adaptive nature of extremophiles and underscores the importance of defining life’s true upper and lower limits. The combined effects of multiple stressors reveal not only biotechnological innovations but also inform astrobiological models for habitable zones beyond Earth [12, 13].

In addition to naturally occurring extreme environments, engineered systems can expose microorganisms to stress conditions that are just as severe and at times more complex. Spaceflight-associated habitats, including the International Space Station (ISS) and space mission–associated environments including spacecraft assembly facilities (SAFs), represent some of the most stringently controlled and biologically challenging environments ever studied. These habitats function as polyextreme habitats, imposing chronic stressors such as microgravity, elevated radiation, desiccation, nutrient limitation, oxidative stress, and repeated chemical disinfection. Unlike classical natural extremes (e.g. hydrothermal vents or hypersaline lakes), ISS and SAF environments are engineered extremes, where microbial persistence occurs under continuous human occupation and aggressive contamination control. Over the past 25+ years, systematic microbial surveillance and cultivation-based programs have demonstrated that these environments harbor rare but viable extremophiles, many of which are poorly represented in terrestrial reference databases [14–17]. Μicroorganisms associated with the ISS and spacecraft assembly cleanrooms represent a unique class of high-stress survivors shaped by microgravity, radiation, desiccation, and stringent decontamination [18]. Genome-resolved investigations of space-associated taxa have uncovered novel biosynthetic gene clusters, antimicrobial resistance determinants, and stress-adaptation systems that are rare or absent in terrestrial reference sets, broadening the landscape of functional and chemical space. Leveraging large culture collections and integrative data analytics allows predictive discovery of bioactive compounds and resistance mechanisms [19].

Due to the environments in which extremophiles reside, they have evolved distinctive amino acid compositions and protein structures. Such unique changes have been observed in the most prominent extremophiles categories, thermophiles, psychrophiles, halophiles, and piezophiles.

In thermophiles, proteins are often stabilized by increased hydrophobicity, forming larger hydrophobic cores, more disulfide bonds, and a greater number of ionic interactions. These features reduce protein flexibility and raise the melting temperatures, ultimately preventing denaturation at high temperatures. In contrast, psychrophiles follow the opposite trend. Their proteins are more flexible due to fewer stabilizing bonds, such as salt bridges and hydrogen bonds, while their amino acid composition favors smaller and/or polar residues. By incorporating extra surface loops alongside lower proline content, psychrophiles can avoid protein backbone solidification and maintain catalytic activity at low temperatures. On the other hand, halophiles are characterized by protein surfaces enriched in acidic amino acids, which allow them to remain soluble and functional in high salt concentrations. Interestingly, in many halophiles, proper protein folding and catalytic function can only occur at relatively high salt concentrations, which is further aided by reduced hydrophobicity and, occasionally, by the insertion of small loops that increase flexibility [20, 21].

The conserved and well-characterized metabolic enzyme malate dehydrogenase (MDH) was used to examine whether the predicted amino acid and protein structural adaptations in extremophiles are also observed in this protein [22]. Thus, thermophilic MDH was characterized by an increase in large-chain and charged amino acids. This is correlated with a higher number of ionic interactions, which would lead to a higher overall protein stability. Halophilic adaptations were characterized by an increase in acidic, hydrophilic amino acids, accompanied by a decrease in basic amino acids. Psychrophilic adaptations were more challenging to evaluate due to the underrepresentation of MDH in psychrophiles. Despite the limited dataset, psychrophilic MDH was characterized by increased hydrophilic amino acids, which facilitate water coordination, preventing ice formation and ultimately increasing the overall flexibility through internal hydration.

While amino acid analyses can reveal trends of general protein adaptations, a structural analysis can provide deeper insights into how proteins have adapted to their environment. On average, thermophilic MDH structures are larger than their mesophilic counterparts, with only a few exceptions, suggesting that not all adaptations are used universally. Additionally, these structures were found to contain more basic residues on the surface. Conversely, all halophilic structures were smaller, very similar in volume, and included a higher number of acidic amino acids on the surface. The small psychrophile dataset still showed a significant increase in size compared to the mesophilic counterpart. The larger volume might have been caused by the rise in surface cavity and looser folding [21, 23].

In extreme habitats, the protein, amino acid, and structural adaptations mentioned above could affect ecosystem composition at the microbial community scale. These biochemical adaptations enable extremophiles to occupy niches inaccessible to ordinary organisms, thereby shaping the microbial community by excluding organisms lacking niche-specific protein modifications.

At the microbial community scale, niche-specific adaptations among all community members have led to various metabolic dependencies, termed syntrophic relationships. Syntrophy is a cooperative interaction in which two or more microorganisms rely on each other to complete a metabolic process that neither could perform independently. This process is observed primarily in energy-poor environments, such as deep-sea sediments, hydrothermal vents, and hypersaline brines. The main interactions that support syntrophic interactions are hydrogen/formate interspecies electron transfer [24], direct interspecies electron transfer (DIET) [25], electron exchange via intercellular soluble redox shuttles such as quinones and flavins [26], and physical electron conduction through nanowires and vesicle-like appendages [27].

A fascinating example of a syntrophic relationship occurs in anaerobic methane oxidation, where anaerobic methanotrophic archaea (ANME) and sulfate-reducing bacteria (SRB) persist in deep-sea hydrothermal vents up to 60°C and in cold marine seeps. ANME can activate and oxidize methane via a reversed methanogenesis pathway, in which SRB must scavenge and reduce the intermediate products. This reaction is thermodynamically feasible only if the SRB performs the reduction reaction immediately. Due to the limited energy available in these extreme environments, DIET is more favorable for interaction between the two syntrophic partners [27].

Since their discovery, extremophiles have revealed a vast and largely underexplored reservoir of resources that could be of great benefit to biotechnology. Their long evolutionary history under extreme physicochemical conditions has allowed for the development of specialized adaptive mechanisms, enabling them to exploit the limited available resources utilizing unique enzymes, biomolecules, and secondary metabolites. As a result, extremophiles have attracted significant interest in biotechnology, contributing to the sustainability of industrial processes and supporting the transition toward a greener, bio-based economy [28]. Moreover, they have also been widely applied in bioremediation where they reduce, transform, immobilize, and detoxify contaminants in disturbed environments [29]. Across several sectors, extremophiles facilitate demanding industrial reactions using extremozymes. Their remarkable stability allows them to function under harsh conditions incompatible with conventional enzymes [9, 30]. Furthermore, the conformational resilience, flexibility, and overall robustness of extremoenzymes show promise in preventing protein misfolding and aggregation associated with neurodegenerative and cardiovascular diseases [31]. Extremophilic *Actinobacteria* have been found to produce enzymes and secondary metabolites with potent antibacterial activity against multidrug-resistant pathogens, as well as antifungal, anti-Human Immunodeficiency Virus (HIV), anticancer, and anti-inflammatory compounds [32]. Beyond their potential for biotechnological applications, the study of extremophiles can enhance our understanding of terrestrial analogue environments, refine hypotheses on the origins of life, and illuminate the evolutionary and physiological boundaries that define life on Earth [33–35]. Finally, since extremophiles were among the first pioneers of life on our planet, they remain the most promising astrobiological model organisms for pursuing extinct or extant life in extraterrestrial environments [36–39].

In this review, we first cover the evolution of methods for discovering extremophiles, ranging from classical cultivation and *in situ* techniques to advanced biomolecule extraction and multi-omics workflows. We then catalogue the critical bioinformatics resources, datasets, and databases for interpreting the (meta)genomics of extremophiles and conclude with the transformative potential of machine learning (ML) and deep learning.

## Field sampling and cultivation strategies

### In situ

**Figure 2:**
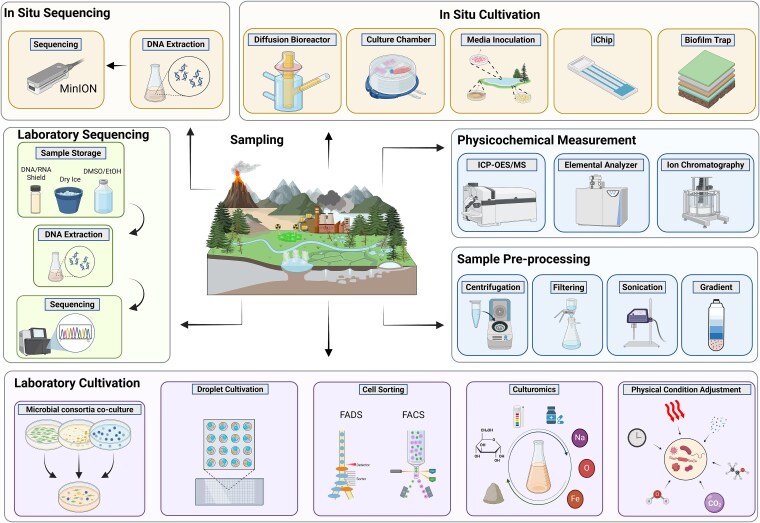
*In situ* and laboratory techniques for the cultivation and sequencing of extremophiles. Microbial cultivation and analysis can be performed either directly in the native environment or after samples are transported to the laboratory. *In situ* approaches include cultivation systems and nanopore-based sequencing. Physicochemical measurements and sample preprocessing provide information on sample composition and help reduce contaminants. DNA extraction and sequencing can be conducted in parallel with cultivation or independently, generating large-scale genetic and metabolic datasets that characterize native microbial communities. Laboratory cultivation strategies range from simple enrichment to advanced methods such as co-culture, droplet-based cultivation, fluorescence-activated cell sorting, and culturomics.

Extremophiles often require complex growth conditions, including interactions with other microbes and habitat-specific nutrients. Therefore, attempts to isolate them under standard laboratory conditions frequently result in the loss of many, if not all, novel extremophiles. *In situ* cultivation, although lacking the precision and control of laboratory setups, can provide access to extremely delicate extremophiles that cannot survive outside of their environment or have complex metabolic relationships with other members of their local microbial community (Fig. 2).

The process from sampling to assembly begins with environmental sampling. In the case of extremophiles, sites are targeted based on their extreme conditions and physicochemical parameters. Geothermal hot springs, hypersaline lakes, acidic rivers, highly alkaline soils, and nuclear-contaminated sites are among the extreme environments [7].

Before sampling, efforts to cultivate and capture extremophiles can take place at the location of interest. Solid and liquid media can be prepared in advance using both nutrient-rich and nutrient-poor formulations. This process can be enhanced by using portable culture chambers. By using these designs, physical conditions such as temperature, pH, and salinity can be carefully adjusted to further enhance the cultivation of fastidious microorganisms. In addition to controlled *in situ* inoculation under adjusted conditions, microbes can also be cultivated using the exact native environmental factors present at the site.

Diffusion chambers and biofilm traps are efficient and widely used methods for growing and capturing microbes directly in their natural environments. The most common diffusion systems are diffusion bioreactors and the iChip. The bioreactors are relatively large chambers with semipermeable membranes that allow microbes to grow, often in bulk, under near-natural conditions. This method enables microbes to maintain metabolic crosstalk, making it particularly useful for studying community dynamics and syntrophic relationships. The iChip employs a similar concept but is designed to contain hundreds of tiny wells on a microfabricated plate. Compared with bioreactors, this device promotes the growth of single, isolated microbial lineages, providing them with their own nutrient stream from the environment [40]. Biofilm traps, in contrast, are sterile abiotic surfaces (rocks, glass, steel, ceramics, or minerals) deployed directly into the environment. Although less complex, they offer an interesting cultivation method that targets *in situ* natural colonization by free-living microbes. This technique is mainly used to study early colonizers, biofilm-producing microbes, and microbe–substrate interactions under natural conditions [41].

Finally, *in situ* sequencing can also be performed using the well-established MinION nanopore technology, which not only enables immediate identification of novel taxa, genes, proteins, and metabolic pathways but also preserves the integrity of fragile DNA and RNA that would otherwise degrade during transport.

### Cultivation

Cultivation in the laboratory can still yield significant isolation success when conducted rapidly (Fig. 2). Using only fresh samples, a few hours old, and knowing the site’s physicochemical composition beforehand can significantly increase cultivation efficiency. Retrieved samples can include sediment, water, biofilm, or rock and should always be collected using sterile equipment and aseptic techniques to prevent non-native microbial contamination. The choice of sample storage depends on the downstream experiment. For cultivation purposes during the expedition, samples can be stored on ice or at ambient temperature. For sequencing, samples should be kept on dry ice and prepared in a nucleic acid stabilization solution when available [42].

To obtain fastidious microorganisms, recent efforts have focused on enhancing the culturable fraction by optimizing culture conditions. Strategies include refining physical parameters (temperature, pH, oxygen concentration, incubation time), adding selective or stimulatory compounds (e.g. antimicrobials, extracellular metabolites, pollutants, signaling molecules), applying single-cell sorting technologies and microfluidic cultivation, and designing synthetic microbial consortia with both native and non-native supportive microbes. Minor yet crucial adjustments of media composition, based on measured physicochemical parameters at the source environment, have also been shown to significantly improve microbial recovery [43].

Adjusting the physical and chemical conditions of culture media (temperature, pH, salinity, pressure, or unusual substrates) helps recreate the harsh, niche-specific conditions required by certain extremophiles [21]. Mimicking these extremes provides the selective pressure needed for extremophiles to grow where standard media would prove to be inefficient.

Another significant factor is the presence of “helper” microbes. Microbial co-cultures can provide essential metabolites, detoxify the environment, and generate gradients strictly required by many syntrophy-dependent extremophiles. Tweaking physicochemical conditions and using co-cultures have resulted in substantial improvements in laboratory cultivation, and these can be further enhanced with microfluidic and cell-sorting approaches. Through droplet cultivation, microdroplets containing varying nutrients or conditions can encapsulate single microbial cells. This method can generate up to millions of parallel microscale incubators. This approach is analogous to the iChip, where each droplet forms a separate environment that allows slow-growing extremophiles to escape competition from fast-growing taxa [40, 44, 45]. Cell-sorting techniques such as Fluorescence-Activated Cell Sorting (FACS) and Fluorescence-Activated Droplet Sorting (FADS) can physically separate single cells based on fluorescence signals into wells or droplets. These methods enable precise single-cell isolation from complex samples. They can specifically enrich cells that show activity under stress conditions such as exposure to UV, high salinity, or heavy metals [46, 47].

Additionally, live/dead staining can be used to separate extremophil-es from nonextremophiles. Other mechanisms include adding fluorescent substrates or probes linked to extremophile-specific processes (e.g. sulfate reduction, compatible solute production) and stains that bind to protective molecules extremophiles produce under stress. Together, these act as filters to enrich extremophiles [48, 49].

Lastly, integrating multiple media formulations, physicochemical conditions, and incubation times can yield hundreds of combinations, enabling high-throughput culturing. Traditional culturomics does not require sequencing before cultivation. However, genome-informed culturomics significantly enhances the success rate by using sequencing data to design tailored media that compensate for missing pathways and supply essential metabolites. With this approach, the odds of culturing previously uncultivable extremophiles significantly increase (Fig. 2).

### Biomolecule extraction

Once extremophile samples are collected and isolated, biomolecules such as DNA, RNA, proteins, and metabolites can be extracted for genomics, transcriptomics, proteomics, and metabolomics, respectively.

The initial step in all procedures involves cell lysis, achieved by the mechanical or chemical disruption of the cell wall. DNA can be isolated using silica-based columns, magnetic beads, or organic extractions followed by alcohol precipitation and phenol–chloroform separation. Next, purity and concentration can be assessed using spectrophotometric measurements and fluorometric assays, while fragment size distribution and integrity are more accurately evaluated with capillary electrophoresis systems [50].

DNA extraction from extremophiles generally follows the same routine protocols and kits used for other microbes. However, protocol modifications have been shown to improve the yield and quality of extremophile DNA. Many extremophiles, particularly archaea and spore-forming bacteria, possess robust cell envelopes [51], inhabit highly inaccessible niches (e.g. thermophiles in biofilms, halophiles within salt crystals) [7], and are often in the presence of inhibitory substances that can co-purify with DNA and interfere with downstream applications [52].

To improve the recovery of high-molecular-weight (HMW) DNA suitable for long-read sequencing, several adaptations can be employed. These include extended enzymatic lysis, the use of protective additives [53], gentle mechanical disruption [54], and agarose plug methods [55]. These modifications, either in place of or in combination with traditional DNA extraction techniques, have been shown to increase DNA yield and fragment size while reducing shearing.

Additionally, selective lysis strategies can enhance DNA recovery from diverse cell types. Tailored enzyme cocktails can be used to ensure comprehensive cell lysis (e.g. MetaPolyzyme) [56], and lysis conditions can be adapted to exploit natural vulnerabilities, such as inducing autolysis in halophilic archaea through osmotic shock [57]. Indirect extraction methods, in which cells are first pelleted and then lysed in a controlled buffer, can also reduce the co-extraction of inhibitors [58]. In exceptionally resilient taxa, such as deep-sea archaea, hot alkaline lysis has been employed effectively to recover DNA from otherwise inaccessible cells [59]. Environmental samples from extreme habitats often contain inhibitory substances, such as humic and fulvic acids, or high salt concentrations, which can co-purify with DNA and interfere with polymerase chain reaction (PCR), library preparation, and sequencing. To avoid or limit this, additional steps can be incorporated. These include pre-extraction washing and sterilization (e.g. surface sterilization of halite crystals with hydrochloric acid and bleach), desalting and concentration via size-exclusion chromatography [60], and the use of detergents and precipitation agents such as cetyltrimethylammonium bromide, to remove humic substances [61]. The implementation and refinement of these strategies will continue to expand our understanding of extremophilic communities and our ability to study life in Earth’s most challenging environments. After nucleic acid extraction, standard library preparation protocols [62] can be followed to proceed to sequencing. Once sequencing data are obtained, they can be processed for a wide range of downstream analyses, including assembly, annotation, and comparative genomics.

Protein extraction requires solubilization with detergents or chaotropic agents, followed by clarification through centrifugation or with various precipitation techniques. This is then followed by their extraction using mixtures of methanol, acetonitrile, chloroform, or water to capture both polar and nonpolar compounds. A range of analytical methods can be used to confirm the quantity and quality of the biomolecules of interest. Proteins can be quantified using colorimetric assays. Their quality can be verified through immunoprecipitation for specific target recovery and Western blotting for detection of expression and integrity. Metabolites are profiled and quantified by chromatography-coupled mass spectrometry or Nuclear Magnetic Resonance (NMR) spectroscopy.

### Extremophiles in the omics era

In extremophile research, samples are collected from chemically harsh environments, where DNA degradation, contamination, and limited usable sequencing data are common. Shotgun metagenomics is the gold standard for uncovering and characterizing the taxonomic distribution of microbial communities in different environments. Recent advances in sequencing technologies and bioinformatic tools for decontamination, *de novo* assembly, binning, and taxonomic classification have enabled the detection of low-abundance and rare taxa with unprecedented resolution [63, 64].

Depending on DNA quantity, integrity, and environmental chemistry, researchers use short-read sequencing, such as Illumina, to obtain highly accurate, cost-effective data suitable for metagenomic surveys and taxonomic profiling, though these methods often produce fragmented assemblies. Conversely, long-read sequencing technologies, including Oxford Nanopore Technologies (ONT) and PacBio, generate reads spanning several kilobases, enabling improved genome assembly and the resolution of repetitive regions, plasmids, and structural variants. The ONT MinION platform, which performs both barcoding and shotgun metagenomic sequencing, is ideal for extreme environments where the microbial community is sensitive and can provide immediate feedback on sample quality and community composition. However, if the MinION platform is unavailable and higher sequencing accuracy is desired, established laboratory sequencing platforms are used. In challenging environments where DNA fragmentation or low biomass limits yield, hybrid sequencing approaches that combine long- and short-read data can maximize assembly continuity and accuracy [65]. Targeted sequencing approaches, such as 16S ribosomal ribonucleic acid (rRNA) gene amplicon sequencing on Illumina MiSeq or Ion Torrent platforms, are also widely used in extremophile studies to characterize microbial community composition when DNA yield is low or reconstruction is impractical [66].

Following sequencing, strict quality control (QC) thresholds and host decontamination are essential to remove bias. To achieve reliable downstream analyses, quality control is performed on raw sequencing data first. Then reads are mapped to reference host genomes using alignment tools to remove contaminant DNA. Following preprocessing, high-quality reads are assembled into contigs to reconstruct longer genomic fragments, which are then grouped into metagenome-assembled genomes (MAGs) using binning algorithms [67]. Given the low biomass of the microbial community of interest, the ideal approach is a hybrid strategy combining long and short reads. However, depending on the environment’s severity, fragmentation of the total DNA can be high, limiting sequencing to short reads. The completeness and reliability of MAGs are evaluated, and then dereplication is performed to generate a nonredundant set of high-quality genomes. MAGs and isolated genomes are then taxonomically classified using a variety of pipelines and viewers [68, 69]. Following metagenomic assembly and recovery of MAGs, functional insights are gained through genome annotation and multi-omics approaches, which provide the functional and evolutionary context needed to interpret extremophile adaptation [70].

Genome annotation integrates multiple reference resources to assign functions to predicted genes. The first step of the workflow is protein prediction. This process identifies potential protein-coding regions, known as Open Reading Frames, on MAGs or scaffolds using tools such as Prodigal. The next step is to identify and functionally characterize the coding sequences, as well as transfer RNAs (tRNAs) and ribosomal RNAs (rRNAs). This step is particularly challenging in extremophile research because many genes encode highly divergent proteins with few homologs in existing databases. Functional assignment can be achieved by tools that perform sequence similarity searches against different databases. Still, in this case, annotation relies more heavily on hidden Markov model profile searches, which detect conserved protein domains and motifs [71, 72].

Once functionality is established, genes are mapped to known metabolic pathways and enzyme classifications, resulting in the reconstruction of metabolic pathways. This process highlights metabolic traits essential for surviving in extreme environments, such as stress tolerance, energy acquisition, or the production of extremozymes. It thus provides insight into the functional potential of extremophilic organisms. To place MAGs in a phylogenetic context, first conserved marker genes are identified and aligned, followed by multiple sequence alignment. Phylogenetic trees are then constructed using model selection to optimize accuracy and can provide insights into evolutionary relationships, horizontal gene transfer, and the identification of novel lineages. This type of analysis of extremophile MAGs often uncovers deeply branching lineages, lateral gene transfer events, and novel taxa, expanding our understanding of microbial diversity in extreme ecosystems [62].

**Figure 3:**
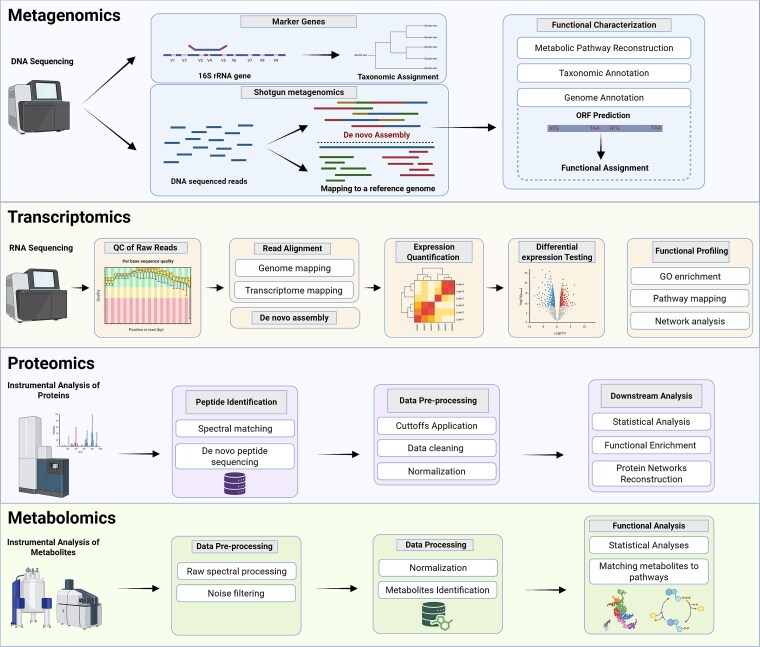
Multi-omics workflows for extremophile research. Metagenomic data can be processed either through shotgun metagenomics, enabling *de novo* assembly and genome-centric analyses, or through rRNA marker gene sequencing for taxonomic assignment. Reconstructed genomes are then subjected to functional annotation, metabolic pathway reconstruction, and phylogenomic analysis. Transcriptomics begins with RNA sequencing followed by read quality control, alignment or *de novo* assembly, expression quantification, differential expression testing, and functional profiling through GO enrichment, pathway mapping, and network analysis. Both proteomics and metabolomics are based on mass spectrometry. Metabolomic analysis includes spectral data pre-processing, metabolite identification, and functional analysis. Proteomics includes peptide identification, data preprocessing, and downstream statistical and functional analyses to characterize protein abundance and changes.

Unlike shotgun metagenomics, which sequences all genomic material and often involves complex genome assembly, 16S rRNA gene amplicon sequencing follows a more targeted approach. Specifically, it focuses on amplifying and sequencing a specific, highly conserved genomic region, the 16S rRNA gene, that serves as a barcode for prokaryotic organisms. These data bypass genome assembly entirely; instead, the raw sequencing reads undergo denoising, which outputs a set of amplicon sequence variants. These variants are unique biological sequences that can be resolved down to a single-nucleotide difference and provide strain-level insights into the microbial community [73] (Fig. 3).

Even though metagenomic analyses remain the primary strategy for identifying novel genes and classifying microorganisms in extreme environments, the presence of a gene does not guarantee its expression or translation into a functional protein. Therefore, metatranscriptomics and metaproteomics have emerged as complementary approaches to uncover the functional microbial landscape of extremophiles.

Metatranscriptomics enables the characterization of gene expression profiles of microbial communities, ultimately revealing the molecular cascades underlying stress responses and adaptation mechanisms in extreme conditions. A typical metatranscriptomic workflow includes quality control, trimming (if necessary), decontamination, *de novo* assembly, transcript-level taxonomy, and functional annotation [74]. Moreover, differential expression analysis using standard RNA-sequencing algorithms, such as DESeq2 [75] and edgeR [76], enables researchers to compare gene expression programs across different conditions. Despite their importance, metatranscriptomics remains technically challenging because intact RNA is difficult to isolate from these environmental samples. Nevertheless, they have been implemented in several studies. For instance, metatranscriptomic studies have elucidated the metabolic processes underlying carbon mineralization in active methanogenic communities [77], characterized altered metabolic activity in hydrothermal vents based on the distance from the vent [78], and demonstrated that archaea and bacteria follow distinct osmoadaptation strategies in hypersaline environments [79]. Collectively, these findings highlight the importance of metatranscriptomics as a complementary tool in addressing extremophiles (Fig. 3).

Accordingly, metaproteomics is used in combination with metagenomics to characterize the protein landscape of microbial communities. Samples are processed to extract proteins, which are then digested into peptides and analyzed by mass spectrometry (bottom–up approach). Then, the resulting spectra are matched to appropriate reference databases either from public repositories or based on metagenomic assembled sequences. The identified proteins can be further used for taxonomic assignment, functional annotation, quantification, and differential expression analysis [80]. Similarly, mass spectrometry can be used to profile microbial communities to characterize small-molecule metabolites and the underlying biochemical pathways involved in microbial adaptation to extreme conditions [81, 82].

Metaproteomics offers deeper insights into the functional landscape of microbial taxa, enabling the comprehensive cataloguing of community composition, characterization of the metabolic processes that support survival under extreme conditions, and the discovery of novel enzymes with potential biotechnological applications [64]. For instance, a previous study integrated metaproteomics and metagenomics to characterize the microbial biodiversity of an Andean high-altitude lake [83]. Similarly, metaproteomic analyses revealed unique ecophysiological adaptations in haloarchaea that enable them to efficiently utilize available light and survive under nutrient-deprived conditions in a hypersaline Antarctic lake [84]. In hydrothermal vent systems, metaproteomics have shown that Gammaproteobacteria dominate inactive chimney communities as autotrophic sulfide oxidizers capable of metal-sulfide dissolution via extracellular electron transfer [85]. In contrast, an earlier study applied metaproteomics to map the microbial communities inhabiting actively venting hydrothermal chimneys [86]. Additionally, multi-omic approaches could lead to an exhaustive characterization of extremophiles, as demonstrated for *T. filiformis*, where integrative analyses uncovered multiple thermostable proteins and revealed its adaptation mechanisms at different temperatures [87] (Fig. 3).

### Bioinformatics resources

The workflows and techniques discussed so far are widely used for genome annotation, taxonomic classification, and functional characterization and are inherently dependent on reference databases (Table 1). These resources provide the necessary information to make predictions and assign annotations. They are crucial in extremophile research, where a lack of reference data often leads to genes and their products being characterized as “hypothetical.”

**Table 1:** Genomic, proteomic, and metagenomic databases that support data-driven analyses of extremophiles and their adaptations.

Databases	Description	Last update date	API access	Batch download capability	Access barriers
1000springs [88]	Genomic, geochemical, and microbiome data from over 1000 hot spring samples in New Zealand.	2018	No	No	Authorized access only
AciDB [89]	Acidophile genomes.	2020	No	No	Open access
BacDive [90]	Strain-level database of bacteria and archaea, providing taxonomy, physiology, morphology, environment, growth conditions, and genomic metadata.	2024	Yes	Yes	Free registration required
BacMet [91]	Metal resistance genes.	2023	No	Yes	Open access
BRENDA [92]	Enzyme database with curated data on functions, substrates, kinetics, and organism-specific properties.	2024	Yes	Yes	Open access
BV-BRC [93]	Workspace that includes a database of pathogenic bacteria and viruses’ genomes, transcriptomes, protein features, protein structures, and metabolic processes.	2024	Yes	Yes	Account required
CAMPR3 [94]	Database and predictor for AMP classes.	2015	No	Partial	Open access
CARD [95]	Antimicrobial resistance gene database.	2024	Yes	Yes	Open access
CAZy [96]	Carbohydrate-active enzymes.	2024	No	Partial	Open access
COG/eggNOG [97, 98]	A database that consists of phylogenetically classified proteins based on their evolutionary relationships, which form COGs.	2024	Yes	Yes	Open access
DisProt database [99]	Database for Intrinsically Disordered Proteins.	2024	No	Yes	Open access
Ensembl Bacteria [100]	Browser for bacterial and archaeal genomes.	2024	Yes	Yes	Open access
European Nucleotide Archive (ENA) [101]	Public repository for nucleotide sequencing data.	Continuous	Yes	Yes	Open access
EzBioCloud [102]	16S rRNA database with taxonomic hierarchy.	2024	No	Partial	Subscription required
Gene Ontology (GO) [103]	Assigns functions to the genes by defining classes of gene functions that have specified relations to each other, representing gene products’ properties.	Continuous	Yes	Yes	Open access
GlobDB [104]	Combines and dereplicates over 14 major microbial genome catalogs.	2025	No	Yes	Open access
GTDB (Genome Taxonomy Database) [105]	Standardized microbial taxonomy based on phylogenomics.	2025	No	Yes	Open access
HaloDom [106]	Halophile database.	2024	No	No	Open access
HaloWeb [107]	Genomic and metabolic pathway data for halophiles.	2022	No	No	Open access
ICEberg 3.0 [108]	Integrative and conjugative elements.	2023	No	Yes	Open access
IMG/VR 4.0 [109] and MetaVR [110]	Repository of viral genome and metagenome data.	2025	Yes	Yes	Open access
IND-enzymes [111]	Thermophilic/psychrophilic hydrolytic enzymes.	2021	No	Yes	Open access
INDIGO [112]	Red Sea extremophile genomics with the AAMG pipeline.	2013	Yes	No	Open access
Integrated Microbial Genomes & Microbiomes (IMG/M) [113]	Platform for microbial genome and metagenome data with standardized annotations and comparative analysis tools.	2025	Yes	Yes	Account required
InterPro [114]	Integrates multiple protein signature databases to classify protein families, predict domains, and assign GO terms.	2025	Yes	Yes	Open access
ISFinder [115]	Database of manually curated prokaryotic Insert Sequence elements.	2025	No	Yes with written authorization	Open access
JGI GOLD [116]	Genome and metagenome sequencing projects, and their metadata.	2024	Yes	No	Account required
KBase (DOE Systems Biology Knowledgebase) [117]	Combines data, tools, and workflows for analyzing microbes, plants, and microbiomes.	2026	Yes	Yes	Account required
KEGG [118]	Reference resource hub for assigning genes or gene products to a specific pathway.	Continuous	Yes	Yes	FTP is paid subscription
KAUST Metagenomic Analysis Platform (KMAP) [119]	Platform for taxonomic and functional profiling of microbial communities	2025	No	Yes	Authorized access only
Marine Metagenomics Portal (MMP) [120]	Marine metagenomics data, integrating public datasets and tools for exploration, including taxonomic and functional annotations.	2018	No	Yes	Open access
MarineMetagenomeDB [121]	Metagenomic datasets from marine ecosystems, including metadata like depth, salinity, and geographic coordinates.	Biannually	No	Yes	Open access
metaMicrobesOnline [122]	Integrated platform for comparative genomics, functional and expression analysis with built-in tools.	2011	No	Yes	Open access
MGnify [123]	Taxonomic, functional, and pathway annotations for raw reads and assembled data across diverse environments.	2023	Yes	Yes	Open access
MiBIG [124]	Secondary metabolite gene clusters.	2024	No	Yes	Open access
Microbe Atlas [125]	A global microbial biogeography platform for exploring environmental distributions of taxa based on public sequencing data.	2020	No	Yes	Open access
MicroScope [126]	Microbial genome annotation and comparative genomics.	2025	Yes	Yes	Account required
NCBI GenBank [127]	Reference sequences are maintained and standardized by NCBI.	Continuous	Yes	Yes	Open access
NCBI RefSeq [128]	Archival database of nucleotide sequences submitted directly by researchers, often with varying annotation quality.	Continuous	Yes	Yes	Open access
NMPFamsDB [129]	Novel protein families.	2023	Yes	Yes	Open access
PAZy [130]	Specialized database for plastic-active enzymes	2025	No	Yes	Open access
Pfam [131]	Protein families and domains.	2024	Yes	Yes	Open access via InterPro
PSORTdb [132]	Protein subcellular localization database for bacteria & archaea, helpful in studying adaptation mechanisms in extremophiles.	2021	No	Yes	Open access
SDADB [133]	Functional annotation database of structural domains in proteins.	2018	No	Yes	Open access
SEED [134]	A database that stores annotated genomes produced by the RAST web server, along with manually annotated genomes.	Continuous	Yes	Yes	Optional login
Sequence Read Archive (SRA) [135]	Archive for raw sequencing data.	Continuous	Yes	Yes	Open access
SILVA [136]	Database of rRNA sequences from bacteria, archaea, and eukaryotes.	2026	No	Yes	Open access
TADB 3.0 [137]	Database of bacterial toxin-antitoxin systems with curated loci, families, and functional annotations.	2024	No	Yes	Open access
TEMPURA [138]	Database of thermophilic and psychrophilic protein adaptations.	2020	No	Yes	Open access
TerrestrialMetagenomeDB [139]	Metagenomic datasets from terrestrial environments, annotated with metadata.	2021	No	Yes	Open access
ThermoBase [140]	Thermophile/hyperthermophile database.	2022	No	Yes	Open access
TIGRFAMs [141]	Database of protein family definitions.	2018	No	Yes	Open access on NCBI
Transporter Classification Database (TCDB) [142]	Classifies transporters and supports genome annotation.	2020	No	Yes	Open access
UniProt [143]	A database that stores protein sequences and functional information.	Continuous	Yes	Yes	Open access
VFDB [144]	Virulence factor database.	2024	No	Yes	Open access
WASPS [145]	Plasmid sequence database.	Continuous	No	No	Open access

Database search is a preliminary step in identifying microbial communities found in any environment. The National Center for Biotechnology Information (NCBI) GenBank [127] and RefSeq [128] host nucleotide sequence data and standardized reference genomes, while UniProt [143], InterPro [114], Pfam [131], and TIGRFAMs [141] hold functional annotations of proteins and domains. At the same time, Gene Ontology (GO) [103] provides a functional classification of genes. KEGG [118] houses information about metabolic, signaling, and regulatory pathways, and the Clusters of Orthologous Groups (COGs)/eggNOG database [97, 98] organizes proteins based on evolutionary relationships.

For community-level analyses, IMG/M [113] and MGnify [123] provide data and pipelines for metagenomic data across diverse environments, while JGI GOLD [116] tracks global genome and metagenome sequencing projects. The Sequence Read Archive (SRA) [135] and the European Nucleotide Archive (ENA) [101] store raw sequencing datasets for reuse. TerrestrialMetagenomeDB [139] and MarineMetagenomeDB [121] host metagenomic datasets from terrestrial and marine ecosystems, and the Marine Metagenomics Portal (MMP) [120] provides taxonomic and functional annotations for oceanic datasets. Comparative studies are enriched by tools such as GlobDB [104], which gathers data from over 14 microbial genome catalogs, and Ensembl Bacteria [100], which provides a genome browser for archaeal and bacterial genomes. SILVA [136] and EzBioCloud [102] are resources that support 16S rRNA-based taxonomic profiling, followed by BacDive [90] for detailed strain-level metadata for bacterial isolates. KBase [117] and metaVR [110] further expand functional modeling and viral metagenomic analysis.

MIBiG [124] contains biosynthetic gene cluster data, DisProt [99] is a repository of intrinsically disordered proteins, and NMPFamsDB [129, 146] is a database of novel protein families from microbial metagenomes and metatranscriptomes. PSORTdb [132] and the Transporter Classification Database [142] provide information on protein subcellular localization and membrane transport proteins, respectively.

The need for a specialized database arose from the unique adaptations observed in extremophiles. ThermoBase [140] contains descriptions of thermophilic or hyperthermophilic organisms, whereas TEMPURA [138] stores prokaryotic growth temperatures. Halophile genomic and metabolic data are available in HaloWeb [107] and HaloDom [106], while acidophile genomes are stored in AciDB [89]. The 1000springs [88] repository contains genomic, geochemical, microbial, and physicochemical data derived from hot-spring samples, whereas INDIGO [112] is explicitly focused on Red Sea extremophiles. IND-enzymes [111], BRENDA [92], CAZy [96], and SDADB [133] host not only extremophile genomes but also data on enzymes derived from extremophiles. PAZy [130] is a specialized database that contains only biochemically characterized plastic-active enzymes. Additionally, metaMicrobesOnline [122] is a platform for comparative genomics and functional analyses that includes tools for studying microbial adaptations across a variety of environments, including extreme environments.

Mobile genetic elements and resistance traits greatly influence extremophile resilience. ISFinder [115] and ICEberg 3.0 [108] contain information about insertion sequences, integrative elements, and transposons. Also, antimicrobial resistance and virulence data are available through CARD [95] and VFDB [144], respectively, while BacMet [91] hosts metal resistance genes, and WASPS contains plasmid sequence data.

### The opportunity of artificial intelligence in extremophile research

As outlined above, advances in experimental high throughput “omics” technologies have generated a large volume of data, offering fertile ground for discovering novel biological mechanisms and industrially valuable enzymes. However, extremophile research is hampered by two persistent bottlenecks: most extremophilic organisms remain unculturable, leaving their genomes uncharacterized and underrepresented in current data collections. And even when input material is available, extremophile omics pipelines routinely contend with limited biomass, DNA damage, contamination, and fragmented assemblies. This implies that descriptive techniques and pipelines developed [147] for readily available mesophilic data are unlikely to generalize to extremophiles. Accordingly, the field would benefit from a paradigm shift toward data-driven, predictive models, posing the fundamental challenge of effectively training them when extremophile datasets are sparse and opportunities to leverage information from other biological systems are limited.

A recurring obstacle in extremophile research is the scarcity of high-quality labels: cultivation is difficult, functional assays are expensive, and environmental samples can be low in biomass and noisy. As a result, many tasks of interest (e.g. predicting thermostability/halotolerance, optimal growth conditions, enzyme pH/temperature optima, or mutation effects for extremozymes) operate in a regime where classical train-from-scratch supervised learning is unreliable. Three complementary ML strategies are particularly relevant in these settings:

(i) *Transfer learning*. Large protein language models (pLMs) pretrained on massive sequence corpora provide general representations that can be fine-tuned with relatively small extremophile-specific datasets, improving performance when labeled examples are limited. For example, TemBERTure predicts thermostability class and melting temperature from protein sequences and emphasizes the importance of training diversity for robust generalization [148].

(ii) *Few-shot learning and meta-learning*. When only tens of labeled variants (or a small number of characterized proteins) are available, few-shot methods adapt models to a new target with minimal data. For instance, FSFP improves protein fitness prediction under extreme data scarcity using only tens of labeled mutants [149], a setting that aligns well with extremophile enzyme engineering, where experimental labels are costly.

(iii) *Multi-task learning*. Extremophile datasets are often thin per task (few measurements per enzyme), but related properties co-vary (e.g. stability, kinetics, temperature/pH dependence, and solubility). Multi-task models can share statistical strength across endpoints, improving sample efficiency and calibration under data scarcity. For example, MPEK is a pretrained multitask framework that jointly predicts enzyme kinetic parameters while conditioning on factors such as pH and temperature [150].

Taken together, these strategies provide a practical path for data-scarce extremophile studies: pretrain broadly (self-supervised on sequences/reads), then adapt with transfer learning, specialize with few-shot or meta-learning when labels are minimal, and stabilize learning via multi-task objectives when multiple weakly labeled endpoints exist.

### Machine learning and artificial intelligence in modern biology

ML, a specific subset of artificial intelligence (AI), has become a central paradigm in modern science, fundamentally reshaping how complex biological systems [151] are analyzed and modeled [152]. At their core, ML approaches involve algorithms that identify dependency patterns among measured variables, learn representations, and make predictions directly from data [153], rather than relying on explicit, manually crafted rules. Specifically, ML refers to algorithms that improve task performance by learning from data, typically by optimizing the parameters of neural networks, decision-tree ensembles, or probabilistic models to minimize a loss function in contrast to hypothesis-driven statistical inference, where predefined mechanistic hypotheses guide model formulation, ML methods directly learn complex relationships from data, postponing the interpretation of underlying mechanisms to subsequent analysis. Notably, recent advances have demonstrated that data-driven ML methods can, under certain conditions, uncover causal relationships [154]. This capability enables the model to detect hidden organization in datasets that are too large, multidimensional, or noisy for conventional approaches and human inspection [155]. Throughout this review, we use ‘ML’ to refer to data-driven modeling (including deep and foundation models) and ‘AI’ to refer to broader systems that integrate ML with hypothesis generation, experiment selection, or automation.

Even within the field of neural network research, a wide range of architectures has been developed to capture the hierarchical and relational structure of different biological data modalities (Table 2). For instance, Convolutional Neural Networks (CNNs) extract local features such as sequence motifs or spatial patterns in molecular structures; recurrent and transformer models capture long-range dependencies in genomic or proteomic sequences; and graph neural networks (GNNs) represent the topology of biological systems, from spatially resolved transcriptomic patterns and protein–protein interaction maps to metabolic pathways. These architectures—and hybrids combining their strengths—have enabled remarkable advances in protein-level prediction, linking amino-acid sequences to structural and functional properties through general protein language models such as AlphaFold [157], ESM-2 [193], and ProtTrans [194]. In parallel, application-specific architectures have been applied to variant and property prediction, estimating functions [195] (e.g. DeepFri), as well as features such as thermostability [184] (e.g. ThermoNet) and solubility [196] (e.g. DeepSol) from sequence or structural inputs.

**Table 2:** Machine learning based tools with potential use in extremophile biology.

Tool	Description/use	Type	Input data type	Primary output/prediction	Limitations
AI4AMP [156]	Uses hybrid encoding and CNN to predict AMP activity.	Deep Learning	Protein sequence	Prediction scores, giving the probability of how much a particular peptide contains AMP activity.	Curated training sets required
AlphaFold [157]	Predicts protein 3D structure from sequence using transformer-based models.	Deep Learning	Protein sequence/alignments	3D protein structure	Does not capture conformational dynamics
AMPlify [158]	Identifies AMPs with long-range dependencies.	Deep learning	Protein sequence	AMP probability score	Performance is limited by lack of detailed AMP training data
AMPScanner [159]	Trains on known AMPs to predict novel AMP candidates from protein sequences.	Deep learning	Protein sequence	Sequences of AMP candidates	Sequences with size <50–60aa
Aurora [160]	Associates bacterial genotypes with environmental adaptation via GWAS-based ML.	Machine Learning	Pangenome matrix & phylogenetic distance matrices	Ranked genes based on association score & model performance tables	Usability is still dependent on the R ecosystem and user expertise
CONCOCT [161]	Uses Gaussian mixture models for contig binning from metagenomes.	Machine Learning	Subcontigs	Clustering file assigning contigs into bins, which can be converted into a single file consisting of a MAG	Contigs need to be cut up prior to binning; reads from all samples should be mapped to a single shared assembly
DeepARG [162]	Predicts antibiotic resistance genes from sequence data using deep neural networks.	Deep Learning	Nucleotide or protein sequences	ARGs with high and low probability	Performs better with assembled contigs
DeepBGC [163]	Detects BGCs in bacterial and fungal genomes	Deep Learning	Nucleotide sequences	BGC regions and class predictions	Sequences with length > 5000 bp
DeepEC [164]	Assigns EC numbers to enzymes using deep learning models.	Deep Learning	Protein sequences	EC numbers and statistics	Limited performance on multifunctional or novel enzymes
DeepGO [165]	Predicts protein functions using sequence and interaction networks, incorporating GO hierarchy.	Deep Learning	Protein sequences	GO term predictions	Easier to work on established annotated organisms
DeepGOMeta [166]	Predicts protein functions using sequence and interaction networks, incorporating GO hierarchy.	Deep Learning	OTU table of relative abundance & protein sequences	GO term predictions	Restricted training scope and incomplete benchmarking context
DeepMicrobes [167]	Taxonomic classification for metagenomics	Deep Learning	Reads & nucleotide sequences	Predicted taxon and abundance	Can be used with specific species; otherwise, it requires retraining
EukRep [168]	Separates eukaryotic and prokaryotic sequences.	Deep Learning	Nucleotide sequences	Gene predictions & Eukaryotic bins	Reduced accuracy on short or low-complexity contigs
evoMIL [169]	Predicts virus–host interactions with transformer architectures.	Deep Learning	Protein sequences	Host label and probability scores	Predictions are limited to hosts with sufficient known virus host associations
geNomad [170]	Identifies mobile genetic elements in metagenomic and genomic data.	Machine Learning	Nucleotide sequences	Viral and MGE annotations	May misclassify novel or highly fragmented elements
GLIMMER [171]	Identification of coding sequences within bacterial, archaeal, and viral genomes	Markov models	Nucleotide sequences	Predicted protein-coding genes (CDS)	Accuracy decreases on error prone or highly fragmented sequences, and the method is computationally intensive
GraphEC [172]	Assigns EC numbers to enzymes using deep learning models.	Deep Learning	Protein (enzyme) sequences	Predicted EC numbers, active sites, and optimum pH of the enzyme	Accuracy is constrained by predicted structural quality and available training features
LookingGlass [173]	Context-aware DNA embeddings predict enzyme function, including optimal temperature for extremophiles.	Deep Learning	Nucleotide sequences	DNA sequence embeddings	Requires task-specific fine-tuning and may show domain bias
Macrel [174]	AMP discovery and MIC prediction from metagenomes and genome sequences.	Machine Learning	Protein sequences	AMP probability score	Emphasizes high specificity at the cost of reduced sensitivity
MaxBin2 [175]	Binning using the expectation–maximization algorithm based on sequence composition and abundance.	Machine Learning	Metagenomic assembly and sequencing reads	Bin assignments and characteristics	Contigs should be preferably of 1000 bp or larger
MetaBAT2 [176]	Unsupervised binning using probabilistic distances; suited for complex communities.	Machine Learning	Contigs	Contig clusters and bins/draft genomes	Dependency on multiple samples
MetageNN [177]	Taxonomic classification for metagenomics	Deep Learning	*K*-mer profiles and DNA sequence	Genus-level classification	Sequence length bias
MetaMIC [178]	Detects misassemblies in metagenomic assemblies using deep neural networks.	Deep Learning	Raw reads, contigs	Misassembly identification and corrected assemblies	metaMIC only corrects contigs that can be split into fragments longer than 1 kb.
MetaTransformer [179]	Taxonomic classification for metagenomics	Deep Learning	Raw DNA reads (short-read data)	Genus-level and species-level classification, and abundance estimation	Updateability and memory constraints (increasing *k*-mer increases memory consumption)
PanPhlAn [180]	Pan-genome-based strain-level microbial profiling.	Machine Learning	Raw reads	Gene presence/absence matrix based on the pangenome	Novel genes and species that might not be present in their pangenome database could be missed
PhyloPythiaS [181]	Performs taxonomic binning of metagenomic contigs using sequence composition and ML.	Machine Learning based	Assembled metagenome contigs	Taxonomic label for each contig	Based on assembled contigs longer than 1 kb.
PrDOS [182]	Prediction of natively disordered regions of a protein chain from its amino acid sequence.	Machine Learning	Amino acid sequence	Two-state prediction (order/disorder) and a disorder probability for each residue	The number of sequences in the multiple FASTA-formatted input is limited to 50
SemiBin/SemiBin2 [183]	Metagenomic binning	Deep Learning	Contigs and BAM files consisting of reads mapped to the contigs	Bins	Not optimized for eukaryotic data
ThermoNet [184]	Quantitative prediction of the changes in protein thermodynamic stability caused by single-point amino acid substitutions (3D-CNN)	Deep Learning	Protein 3D structure (either experimental or computational)	Predicted ΔΔG caused by the given mutation compared to the wild-type protein structure	Requires reliable protein structures and is computationally intensive
ThermoProt [185]	Python package to predict the thermostability of proteins (SVM)	Deep Learning	Protein sequences	Prediction of Tm	Trained on specific IMG-derived sequences
Tiara [186]	Classifies eukaryotic sequences from metagenomic assemblies using deep learning	Deep Learning	Nucleotide sequences	Microbial & organelle taxonomy	Classification accuracy decreases for short sequences (<3000 bp)
TolRad [187]	Random Forest Binary Classifier that uses Pfam domains in bacterial genomes to differentiate radiation-sensitive from radiation-tolerant species	Machine Learning	Nucleotide & Protein sequences	Predicts tolerance (D10 > 200 Gy) or radiosensitivity (D10 < 200Gy	Accuracy is limited by a small and taxonomically biased training dataset
tRNAscan-SE 2.0 [188]	tRNA prediction	Machine Learning	Nucleotide sequences (DNA or RNA)	Prediction of tRNA genes and their features and predicted structure	Accuracy declines for highly atypical or lineage-specific tRNAs and for distinguishing functional genes from pseudogenes without additional contextual data
VAMB/AAMB [189]	Variational autoencoder–based tool for contig binning; uses deep learning	Deep Learning	Nucleotide sequences (contigs)	Contig bins (and optionally taxonomy)	May struggle with low-abundance or highly similar genomes
ViraMiner [190]	Classifies viral contigs using CNN and Long Short-Term Memory layers.	Deep Learning	Metagenomic contigs	Output a value between 0 and 1 indicating the probability of the sequence coming from a viral origin	Performance may decline for highly divergent or novel viruses
Whokaryote [191]	Separates eukaryotic and prokaryotic sequences using sequence signatures	Machine Learning	Metagenomic contig or genomic sequence.	Contigs headers that have been classified as Prokaryotic or eukaryotic in separate files	Contigs with <2 genes or shorter than 5000 bp are not classified
XenoBug [192]	Application of machine learning–based methods to identify novel bacterial enzymes capable of degrading a wide range of xenobiotics	Machine Learning	PubChem ID or an sdf file of the query molecule	Predicted enzymes that can degrade the input molecule, their metagenome source, and pathway information, as well as the Tanimoto threshold at which EC numbers were predicted	Class imbalance among EC enzyme categories restricts training data representation

Another significant contribution of ML lies in integrating multi-omics data. Integrative ML frameworks can learn joint embeddings that map heterogeneous data types into shared latent spaces, uncovering hidden correlations between molecular layers and environmental gradients. A key idea is the combined use of genomic, transcriptomic, proteomic, and metabolomic measurements to construct a virtual cell. This unified computational representation captures cellular state and function as closely as possible [197].

A significant recent development is the replacement of task-specific tools and pipelines by large foundation models. Foundation models are large-scale neural networks trained on vast and highly diverse datasets with multiple objectives to learn generalizable representations that can be adapted to a wide range of downstream tasks with minimal fine-tuning [198]. Genomic foundation models (gFMs) have emerged as a new paradigm for a wide range of applications. Models such as DNABERT-2 [199], Nucleotide Transformer [200], and HyenaDNA [201] have demonstrated broad utility across biological domains, including gene expression and splicing regulation, variant-effect prediction, promoter and enhancer classification, and noncoding variant prioritization. The recently published AlphaGenome model [202] predicts thousands of functional genomic tracks for up to 1 Mb DNA sequence windows and reports strong performance on variant-effect benchmarks. The obtained embeddings provide a versatile foundation for downstream tasks, often outperforming task-specific models in low-data settings. Moreover, pretraining on a large number of genome sequences ensures that models can generalize beyond what they have seen during training.

Recent advances in explainable AI (XAI) further strengthen the synergy between computational modeling and experimental validation [203, 204]. By interpreting the internal structure of models, identifying which residues, motifs, or pathways contribute most to predictions, researchers can generate testable hypotheses and guide targeted experiments [205]. This interpretability is particularly critical in biological systems, where mechanistic transparency determines scientific credibility.

Similar to most other fields in life sciences, metagenomics has seen rapid adaptation of deep learning techniques [206], enabling automated binning, annotation, and element classification across complex microbial communities. VAMB [189] uses a variational autoencoder framework to integrate sequence composition and co-abundance information to perform highly accurate metagenomic binning, recovering 29%–98% more near-complete genomes compared to baseline methods, and even from low-abundance species. Complementing such binning approaches, CheckM2 [207] employs deep neural networks to estimate genome completeness and contamination, improving quality assessment over heuristic marker–based metrics. Beyond assembly, several ML tools address functional annotation and element identification. DeepEC [208] predicts enzyme commission (EC) numbers directly from protein sequences using convolutional networks. On a set of held-out sequences, DeepEC achieved the highest precision/recall (0.920/0.455) compared to five baseline methods. DeepARG [162] classifies antibiotic resistance genes from environmental metagenomes with reported performance exceeding 97% precision and 90% recall across 30 resistance categories. Similarly, DeepBCG [163] identifies biosynthetic gene clusters in bacterial and fungal genomes, accelerating the discovery of secondary metabolites. For mobile genetic elements, PlasFlow [209] uses neural-network classifiers to distinguish plasmid DNA from chromosomal DNA with reported performance up to 96% classification accuracy, 85.98% recall, and 72.17% precision, and DeepVirFinder [210] detects viral sequences directly from assembled contigs, achieving Area Under the Receiver Operating Characteristic Curve (AUROC) scores between 0.93 and 0.98 depending on contig length. The recently proposed ViraLM [211] uses the DNABERT-2 foundation model architecture to improve the identification of viral sequences with low similarity to known viruses and performs robustly on fragmented metagenomic assemblies, which have been challenging for prior methods. In benchmarking, ViraLM achieved precision comparable to protein-based baseline tools while maintaining higher recall across host groups. Notably, it attains F1-scores of 0.91 and 0.92 on distinguishing viruses from bat- and human-origin contigs, respectively, while the evolutionarily closest annotated sequences in the training dataset are from insects.

A complementary trend is the move toward reference- and assembly-free inference directly from metagenomic reads. For example, the REMME DNA language model and its fine-tuned version REBEAN predict enzyme-class annotations for short sequencing reads [212], consistently identifying three- to six-fold more enzymatic reads compared to alignment-based tools. This is especially valuable when extreme-environment samples yield low biomass, fragmented DNA, or highly fragmented assemblies.

Together, these models exemplify how deep learning architectures can capture nonlinear patterns in high-dimensional genomic data, yielding more accurate and scalable predictions than traditional rule-based or alignment-dependent pipelines.

While the methods above illustrate the power of ML, their application to extremophile datasets raises reliability concerns because available training data are sampling-, cultivation-, and annotation-biased and can differ markedly from the target domain. Two broad failure modes are therefore important to consider: Large Language Model (LLM)/agentic tools used for literature synthesis or automated reporting can generate unsupported statements (“hallucinations”) unless outputs are grounded in traceable sources, whereas sequence/structure foundation models more often fail through overconfident extrapolation and bias amplification under domain shift, producing plausible, high-confidence predictions that reflect training-set correlations rather than true mechanistic determinants. To promote valid and reproducible use, we recommend: (i) careful dataset preparation including metadata and train–test split criteria, as well as bias documentation, (ii) domain-shift–aware training and evaluation (e.g. leave-one-clade/biome-out testing and leakage controls), (iii) uncertainty calibration with abstention and triangulation across complementary methods, and (iv) experimental validation. Accordingly, AI/ML outputs should be treated as hypothesis-generating and require orthogonal validation before guiding further decisions.

### Machine learning in extremophile research

#### Predicting protein and enzyme adaptations

Adaptive traits often arise through alterations of existing proteins rather than the evolution of entirely novel sequences and functions. Comparative analyses have shown that extremophilic proteins maintain canonical folding patterns while incorporating stabilizing features. Alterations in amino acid composition, increased surface charge density, strengthened hydrophobic cores, or additional salt bridges enhance resilience under harsh conditions. These principles are well illustrated in studies of thermophilic and halophilic enzymes, such as α-amylases and proteases, where subtle sequence variations confer substantial improvements in thermostability and solvent tolerance [213–215].

ML methods now offer systematic ways to generalize and predict such adaptive molecular properties directly from sequence or structure. Models trained on large protein datasets have achieved accurate predictions of thermostability [216], halotolerance [217], and acid stability [218], demonstrating ML’s capacity to learn nonlinear relationships between residue composition and environmental performance. One recent example, the thermostability prediction approach TemBERTure [219], emphasizes the careful construction of the training dataset with respect to sequence diversity, which is particularly relevant when transferring to underrepresented extremophile lineages. Benchmarking of TemBERTure yielded a comparable F1 score of 0.9 for the prediction of protein thermal class.

**Figure 4:**
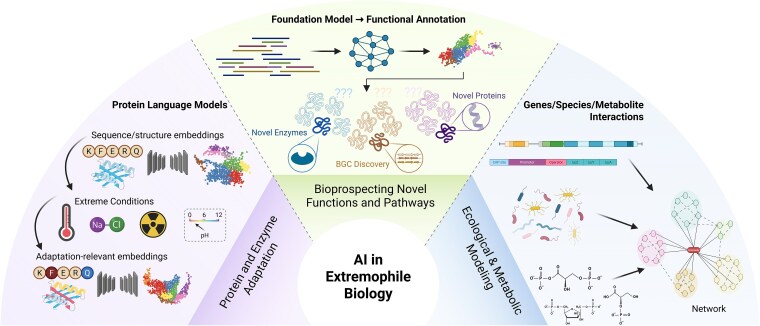
ML in extremophile biology. Protein language models capture sequence and structure features relevant to adaptation under extreme conditions, enabling the prediction of thermostability, halotolerance, and other traits. Foundation models support functional annotation of poorly characterized extremophile genomes, including the discovery of novel enzymes and biosynthetic gene clusters, and integrative AI frameworks model gene-species-metabolite interactions, revealing ecological organization and metabolic exchanges in extreme environments.

Furthermore, pretrained protein language models such as the ESM family or ProtT5 can be used in a transfer learning setting by fine-tuning on extremophile datasets to capture adaptation-relevant sequence embeddings, indicating which sequence patterns or global features correlate with environmental adaptation, thereby helping to identify, e.g. molecular determinants of stability without requiring explicit structural data. This approach can help overcome limited data availability for extremophilic organisms by leveraging the extensive general protein knowledge in foundation models (Fig. 4).

#### Bioprospecting novel functions and pathways

In addition to adaptations in existing proteins, some extreme environments required the innovation of new protein functions. The systematic exploration of such biomolecules and biocatalysts, known as bioprospecting, has become a central task in extremophile research. Both function-based and sequence-based metagenomic strategies have enabled the discovery of novel genes and enzymes directly from environmental samples, thereby overcoming the cultivation bottlenecks inherent to many extremophilic microorganisms [220]. gFMs have already been adapted in recent studies to model metagenomic sequences to provide informative DNA embeddings for segregating species and discovering multigenic biosynthetic gene clusters [221], as well as to improve the identification of coding regions from fragmented and mixed DNA sequences [222]. To link enzymatic function to ecological context, another recent report, XenoBug [192] uses random forests to predict the EC numbers of bacterial enzymes capable of biodegrading environmental contaminants. However, a significant limitation persists: a substantial fraction of recovered sequences shows no detectable homology to known proteins, rendering standard homology-based function-prediction approaches inapplicable. This has motivated method development beyond general property prediction, using deep learning architectures to classify enzyme function and predict catalytic residues. Tools such as DeepEC [208], DeepEC-Transformer [164], and GraphEC [172] combine convolutional, transformer, and GNN approaches to predict EC numbers and active sites from sequence and structural features. Importantly, these methods often generalize well even when trained predominantly on nonextremophilic data, benefiting from the broad representational power of deep learning models. Looking ahead, multimodal protein foundation models fine-tuned on extremophile datasets may further accelerate exploration of this functional “dark matter” (Fig. 4).

#### Modeling ecological and metabolic interactions

Extremophilic organisms often form complex, interdependent microbial communities whose collective metabolism enables survival under otherwise prohibitive conditions. Understanding the ecological and metabolic interactions can therefore be essential for understanding how energy, nutrients, and stress-response mechanisms are linked among community members. A previous study [223] employed machine learning to predict syntrophic and metabolic interdependencies among thermophilic consortia, revealing cooperative nutrient exchange and redox coupling strategies that sustain community function at high temperatures.

More generally, network-based modeling approaches could be used to model microbial community structures and functional organization across multiple scales [224]. GNNs [225] can represent species, genes, or metabolites as nodes connected by learned interaction edges, enabling the prediction of emergent community properties such as stability and resilience. Such integrative models offer a data-driven path to understanding how extremophile ecosystems maintain homeostasis (Fig. 4).

## Discussion

Through long-term adaptation, extremophiles have evolved resistance mechanisms that allow them to thrive in some of the most inhospitable environments on Earth, including high temperatures, acidity, salinity, pressure, and radiation. These microorganisms have not only enhanced our understanding of microbial biology and the limits of life but also represent a significant untapped source of metabolic and enzymatic diversity. The earliest discoveries in the field of extremophiles led to the identification of essential biomolecules, such as ‘*Taq* polymerase’, enabling the development of revolutionary technologies. The comprehensive characterization of extremophiles will reveal an unexplored pool of biomolecules with promising biotechnological potential. Several studies have already started exploiting the unique characteristics of the extremophiles.

One of the most widely used biotechnological applications regarding enzymes derived from extremophiles is the development of VENUCEANE™. This active ingredient is used in the cosmetics industry, in creams, serums, and gels for the prevention and treatment of infrared-related skin damage. It originates from the extremophilic bacterium *T. thermophilus* strain GY1211. This thermophilic microorganism was first discovered in deep-sea hydrothermal vents and was cultivated under fermentation conditions engineered to yield a thermostable ferment extract rich in enzymatic antioxidants and extremozymes. The resulting ingredient retains activity at elevated temperatures and under solar exposure, making it highly effective against infrared-A- and ultraviolet-induced skin damage [226].

In the bioremediation field, a case of extremophile-based innovation is the Multi-strain Mixed Microbial Application Process (MMMAP). MMMAP is a microbial consortium comprising hyperthermophilic, barophilic, acidogenic, anaerobic bacteria (notably *Thermoanaerobacterium* sp., *Thermotoga* sp., and *Thermococcus* sp.) specialized in enhanced oil recovery. In this innovation, researchers collected anaerobic formation fluid samples from high-temperature oil wells (70°C–90°C) in Mehsana, Gujarat, India, sampling under strict anoxic conditions. This consortium produces a combination of biosurfactants, organic acids, gases, and solvents that work synergistically to reduce oil viscosity, increase reservoir permeability, and improve hydrocarbon mobilization under extreme heat and pressure [227].

Another significant innovation in extremophile-based bioremediation biotechnology is demonstrated in a study evaluating diesel-contaminated soil near the Brazilian Antarctic Station. Indigenous, cold-adapted hydrocarbon-degrading bacteria, including species of *Pseudomonas*, *Sphingomonas*, and *Acinetobacter,* were isolated and used to create a bioaugmentation consortium tailored to Antarctic conditions. When applied *in situ*, these microbial consortia, along with biostimulation treatments, significantly altered community structure and enhanced degradation of total petroleum hydrocarbons, even at near-freezing temperatures. Bioaugmentation proved especially effective in soils with high diesel loads, highlighting the potential of native psychrotolerant strains for polar bioremediation efforts [228].

Despite growing significance, extremophile research remains in its infancy, with several ecosystems and microbial taxa left unexplored due to technical, logistical, and cultivation challenges. AI enables transforming this new field from an observational science into a predictive and generative framework. The synergy between foundation models, multi-omics datasets, and AI-guided experimentation can reveal adaptation principles innate to extremophiles, serving as natural experiments in robustness and adaptability. Key goals include AI-assisted bioprospecting and strain engineering, improving genotype–phenotype mapping in recombinant systems, and, ultimately, anticipating how overexpressed genes or modules perturb cellular state [229, 230]. Insights into metabolic interactions in extreme habitats may inform the design of synthetic consortia optimized for industrial processes under harsh conditions. Beyond these applications, AI could enable the systematic exploitation of extremophiles for bioremediation and terrestrial analogue research. ML–guided screening of metagenomic and proteomic data could identify stress-tolerant enzymes and pathways involved in contaminant transformation, including heavy-metal resistance and degradation of recalcitrant compounds under extreme physicochemical conditions. At the ecosystem level, AI-driven models may optimize the composition and functional balance of extremophile-based microbial consortia, predicting collective behavior and metabolic interactions in harsh environments. Similar approaches could also be applied to terrestrial analogue studies. In such contexts, AI-assisted environmental and metabolic modeling of extremophile communities inhabiting hypersaline, acidic, or radiation-rich ecosystems (conditions that resemble key geothermal and physical features of Mars) could generate insights relevant both to contaminated environments on Earth and to Mars-analog systems.

Nevertheless, the application of AI and machine-learning methods to extremophile research is not without challenges. Available training data are overwhelmingly dominated by mesophilic organisms, and this may impede the proper modeling of distribution shifts associated with important biological differences in extremophiles, e.g. unique physicochemical signatures. To mitigate these limitations, possible strategies include leveraging transfer learning and fine-tuning to adapt representations learned from mesophilic data, while domain adaptation and physics-informed modeling can explicitly incorporate extremophile-specific constraints, such as enhanced structural rigidity in hyperthermophiles or surface charge enrichment in halophiles, thereby improving robustness beyond purely evolutionary similarity.

Finally, using extremophiles as a stress test for model generalization is attractive, but requires carefully designed benchmarks. These should include edge cases such as short or fragmented contigs and rare taxa, standardized environmental covariates, and held-out sites or time points to detect overfitting. Research community priorities should include curated and versioned benchmark suites with quality thresholds for metagenome assemblies and annotated proteins, prospective challenge tasks (e.g. enzyme property or function prediction), and interoperable repositories linking sequence, structure predictions, and experimental outcomes. These efforts will accelerate the impact of AI on extremophile research while ensuring robust, reproducible advances. Conclusively, combining multi-omic approaches with AI to study extremophiles will help us expand the catalogue of life and understand novel biochemical processes, ultimately fueling biotechnological innovation.

**Key Points**

- Extremophiles exhibit unique physiological and molecular adaptations that enable survival under extreme temperature, pH, salinity, radiation, and pressure conditions.
- Advances in multi-omics technologies, metagenomics, transcriptomics, proteomics, and metabolomics have transformed extremophile research, enabling detailed characterization of uncultured and rare taxa.
- Bioinformatics resources and specialized databases are essential for interpreting extremophile genomes, identifying extremozymes, and exploring novel metabolic pathways.
- Machine learning approaches are increasingly central, supporting functional annotation, prediction of adaptive protein traits, metagenomic binning, and discovery of novel enzymes and biosynthetic gene clusters.
- Integrating artificial intelligence with multi-omics approaches provides a predictive framework for understanding extremophile ecology, adaptation, and biotechnological applications, including industrial bioprocessing and bioremediation.

## Data Availability

This study does not produce or analyze new data.
